# Polycaprolactone/Anthocyanin-Based Electrospun Volatile Amines Gas Indicator with Improved Visibility by Varying Bi-Solvent Ratio: A Case of Intelligent Packaging of Mackerel

**DOI:** 10.3390/foods12203850

**Published:** 2023-10-20

**Authors:** Sang Won Kim, Eun Seol Kim, Byeong Jae Park, Yong Woon Jung, Dong Hwa Kim, Seung Ju Lee

**Affiliations:** Department of Food and Biotechnology, Dongguk University-Seoul, Goyang-si 10326, Republic of Korea; ssonekim@naver.com (S.W.K.); rladmstjf@naver.com (E.S.K.); sos0053@naver.com (B.J.P.); jungyu221@naver.com (Y.W.J.); nana4132@naver.com (D.H.K.)

**Keywords:** volatile amines gas indicator, electrospun nanofiber, poly(ε-caprolactone), red cabbage anthocyanin, acetic acid/formic acid bi-solvent

## Abstract

Electrospun nanofibers have been applied as a new technology for gas indicators in food intelligent packaging. A poly(ε-caprolactone) (PCL)/red cabbage anthocyanin (RCA)-based nanofiber volatile amines gas indicator was developed by applying a bi-solvent of acetic acid (AA) and formic acid (FA) in electrospinning. The visibility of color change was improved from pink to blue, compared to blue to yellow-green, when using a single solvent of AA. The solutes of PCL (12.5, 15, 17.5, and 20%) and RCA (10, 20, 30, and 40%) and the solvents of AA/FA (9:1, 7:3, 5:5, 3:7, and 1:9) were applied in electrospinning under the condition of 12.5 cm, 1.0 mL/h, and 20 kV. The optimal microstructure with the thinnest fiber diameter and constant arrangement without forming NF beads appeared under the 7:3 FA/AA, 15% PCL, and 20% RCA condition. The indicator changed from pink to blue with the values of total color change (Δ*E*) of 10, 14, and 18 when exposed to the saturated gas of ammonia solutions of 8, 80, and 800 mM, respectively. The indicator was stable and unchanged in color for 28 days when exposed to light at room temperature. In the application to mackerel packaging, the built-in indicator changed from pink to purple regardless of storage temperature when the spoilage point was reached.

## 1. Introduction

Intelligent packaging easily provides consumers with information about the quality and freshness of packaged food via built-in indicators and color changes [1,2]. The indicators monitor freshness by changing color due to volatile metabolites, such as volatile amines, CO_2_, and hydrogen sulfide, released due to the growth of microorganisms or chemical changes in the food during spoilage [3]. As the volatile amines (trimethylamine, dimethylamine, and ammonia) accumulate in the headspace of the package of protein-rich foods such as meat and fish, the pH increases, changing the color of the indicator [4,5]. Bromocresol green, chlorophenol red, cresol red, and anthocyanin are commonly used as pH-indicating substances in volatile amines gas indicators [6,7]. Anthocyanin is especially desirable because it is a natural and non-toxic dye [1,7]. There are approximately 600 different types of anthocyanins found in nature. Among them, anthocyanins extracted from red cabbage are commonly used in sensors due to their wide pH range and high stability against heat and light compared to anthocyanins extracted from other natural sources [8,9].

Anthocyanins are typically red or orange under acidic conditions, pink under neutral conditions, and blue under alkaline conditions [9]. To develop anthocyanin-based intelligent food packaging, the anthocyanin is incorporated into a polymeric matrix of polysaccharides, proteins, or other polymers by film-forming methods such as casting, sol-gel, and spray-drying [9,10,11]. While the sol-gel method offers several advantages, such as uniform porosity, optical transparency, mechanical strength, and low chemical reactivity [12], there are problems in that the reaction time is relatively long due to the lack of surface porosity and thickness, and large-scale production is limited. In addition, over time, anthocyanin is decomposed, and the function of the film is lost. To overcome this, nanofiber (NF) films are being developed for intelligent packaging [12,13]. Electrospun NF films offer many advantages in gas detection due to their high surface area and porosity and show fast response times.

Electrospinning is one of the representative methods for producing nanofibers. Nanofibers are obtained by applying high voltage to a polymer solution, ejecting the solution, and solidifying or coagulating it. The nanofibers obtained in this way have a great advantage in that their high surface area due to their porous structure improves the reactivity of the indicator and increases sensitivity [14]. Electrospinning has been applied to the development of anthocyanin-based indicators [15,16]. In electrospinning, the quality of the NF produced (fiber thickness and presence or absence of beads) is determined by the polarity of the polymer, solvent, process conditions (flow rate and applied voltage), and environmental conditions (temperature and humidity) [17]. As polymers, hydrophilic PEO, PVA, starch, and hydrophobic PCL have been applied [14,15]. When electrospun NFs of the hydrophilic polymers are in contact with water, the structure dissolves. Therefore, when used as an indicator for monitoring food spoilage, there is a problem in that the polymer matrix is decomposed due to high humidity during long-term storage [2,18]. To solve this problem, a crosslinking process or hydrophobic polymer that confers the NF film more resistant to moisture is used. Poly(ε-caprolactone) (PCL) is a widely known hydrophobic polymer commonly used for medical applications due to its biocompatibility and slow biodegradability as a semi-crystalline polyester [19,20]. Although PCL is easy to process due to its low melting point and PCL films are suitable for food packaging materials intended for refrigerated food [21], it is difficult to obtain bead-free fibers with nano-sized diameters when electrospinning. In addition, PCL is mainly used by relatively toxic and expensive solvents, such as chloroform, dimethylformamide, tetrafluoroethylene, methylene chloride, dichloroethane, and pyridine, and has the disadvantage of low reproducibility [22]. Studies have been continuously conducted to find the optimal solvent for electrospinning PCL using relatively less toxic solvents, such as acetic acid (AA) and acetone [20]. Among them is a colorimetric film made of PCL/anthocyanin using AA as a solvent that changes from blue to dark blue when the food spoils [2]. The disadvantage of using AA as the single solvent is that the reproducibility of electrospinning is insufficient due to the low electrical conductivity of AA, and beads are formed [23]. Meanwhile, it has been reported that when AA and formic acid (FA) are used together, the bead formation can be suppressed, and the structure of NF can be improved [22,24].

In this study, a bi-solvent of AA and FA was used in the electrospun PCL/red cabbage anthocyanin (RCA) NF film to avoid the use of organic solvents and improve the NF structure. First, the electrospinning condition was optimized for the microstructure and color characteristics of the NF film by varying the AA/FA ratio, PCL content, and RCA content. The color change in the indicator to saturated gas of ammonia solutions of various concentrations and at different temperatures was assessed to determine the endpoint of the indicator. Then, it was applied to the packaging of the mackerel, and the color change in the indicator was compared to the quality of the mackerel. The purpose of using the bi-solvent was to enhance the steady state in the electrospinning process and improve the visibility of the color change in the indicator.

## 2. Materials and Methods

### 2.1. Materials

PCL (Mn = 80 kDa), FA (reagent grade, ≥95%), and 25% ammonia (NH_3_) solution were purchased from Sigma-Aldrich (St. Louis, MO, USA). AA (glacial, 97%) was bought from Samchun Chemicals (Seoul, Republic of Korea). RCA was obtained from ES Food (Gyeonggi, Republic of Korea). Protective fabric (EN 14126, Polypropylene 50%, and Polyethylene 50%) was purchased from 1004Yo (Daegu, Republic of Korea). The mackerel was bought from E-Mart (Goyang, Republic of Korea), with the head, tail, fins, and intestines removed.

### 2.2. Electrospinning

After FA and AA were mixed according to the *w*/*w* ratio of 9:1, 7:3, 5:5, 3:7, and 1:9 to form the bi-solvent mixture, PCL was added (12.5, 15, 17.5, and 20% *w*/*w*) and completely dissolved. Then, RCA (10, 20, 30, and 40%, *w*/*w* of polymer) was added and stirred at 100 rpm for 3 h. The dissolved solution was transferred to a 10 mL syringe and electrospun for 20 min by using a nozzle with a diameter of 0.8 mm and fixing the voltage (20 kV), flow rate (1.0 mL/h), and tip-to-collector distance (12.5 cm) [22]. During electrospinning, the temperature was maintained at 25 ± 3 °C, and the relative humidity was maintained at 30 to 50% using a built-in humidifier.

### 2.3. NF Film Indicator Characterization

#### 2.3.1. Scanning Electron Microscopy (SEM)

After cutting the indicator to 1 × 1 cm^2^ in size, it was coated once with a thin gold layer using an ion sputter coater (SPT-20, COXEM, Daejeon, Korea). A scanning electron microscope (SIGMA 300, Carl Zeiss, Oberkochen, Germany) was used with an accelerating voltage of 5 kV. The diameter of the electrospun NF was determined by measuring about 30 individual fibers using ImageJ 1.53t software (National Institutes of Health [NIH], Bethesda, MA, USA).

#### 2.3.2. Thickness and Porosity

A micrometer (Mitutoyo, Kanagawa, Japan) was used to measure the thickness of the film. Porosity was calculated [23].

(1) $$Porosity=1-\frac{\rho_{apparent}}{\rho_{PCL}}\times 100$$

where *ρ*_PCL_ is the density of PCL (1.145 g cm^−3^) and *ρ*_apparent_ is the average density of the electrospun indicator, which was calculated by using the weight and volume (=thickness × area) of samples of 1 cm^2^ (*n* = 10). Thickness was calculated by measuring each of the five parts of one sample (one in the center, two on the left side, and two on the right side) and calculating the average value.

#### 2.3.3. Attenuated Total Reflectance Fourier Transform Infrared (ATR-FTIR)

Using a Nicolet iS50 ATR-FTIR spectrophotometer (Thermo Fisher Scientific, Waltham, MA, USA), the transmittance of the PCL, RCA, and NF indicators (0.5 × 0.5 cm^2^) was measured in a wavelength range of 500 to 4000 cm^−1^, respectively.

#### 2.3.4. Colorimetric Response to Ammonia

The electrospun indicator was cut to 1.8 × 1.8 cm^2^ in size, and the protective fabric was cut to 2 × 2 cm^2^. The indicator was attached to an adhesive tape (MMM, Saint Paul, MS, USA) of 2 × 2 cm^2^, and then the fabric was attached on top of it. The method for exposing the samples to saturated gas of ammonia solutions was modified based on the method proposed by Yildiz et al. [25]. First, 16 mL of ammonia solution (8, 80, and 800 mM) was dispensed into a 100 mL Erlenmeyer flask, and the inlet was completely covered using a prepared indicator. Next, it was kept in incubators at different temperatures (5, 10, and 20 °C) for 30 min. After, the color parameters of the indicator were measured using a colorimeter (CR-300, Konica Minolta, Inc., Osaka, Japan). The values of total color change (∆*E*) were calculated.

(2) $$∆E=\sqrt{{\left({L_{1}}^{*}-{L_{2}}^{*}\right)}^{2}+{\left({a_{1}}^{*}-{a_{2}}^{*}\right)}^{2}+{\left({b_{1}}^{*}-{b_{2}}^{*}\right)}^{2}}$$

where L* is lightness and ranges from 0 (black) to 100 (white), a* is redness (+) to greenness (−), b* is yellowness (+) to blueness (−), and subscripts 1 and 2 represent the initial and after 30 min, respectively. 

#### 2.3.5. Color Stability of Indicator during Storage before Use

After the prepared indicator was cut to 3 × 3 cm^2^ in size, it was placed in a Petri dish and stored in a temperature and humidity chamber (SJ-503HS, Sejong Scientific Co., Gyeonggi, Korea) at 4 and 20 °C and 50% relative humidity, under light and dark conditions for 28 days. The color of the indicator was measured every week using colorimetry (refer to Section 2.3.4) to confirm the color change.

### 2.4. Application of NF Film Indicator

#### 2.4.1. Mackerel Storage

**Figure 1:**
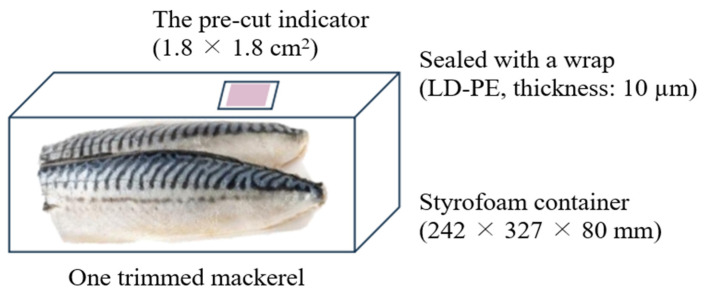
The schematic diagram of mackerel packaging with indicator.

One trimmed mackerel was placed in a 242 × 327 × 80 mm Styrofoam container and the lid was sealed with a wrap (LD-PE, thickness: 10 µm) (Figure 1). 

The pre-cut indicator (1.8 × 1.8 cm^2^) was attached to the inside of the wrap with double-sided tape (refer to Section 2.3.4). The packed mackerel was stored in an incubator at 10, 15, 20 °C, and 50% relative humidity. The color of the indicator was measured every day.

#### 2.4.2. Total Volatile Basic Nitrogen (TVB-N) Measurement

Nitrogenous compounds (ammonia, dimethyl, and trimethyl amine) were measured using the Conway method [26]. After weighing 5 g of stored mackerel (refer to Section 2.4.1), 25 mL of distilled water was added, and the contents were pulverized with a homogenizer (HG-15D, Daihan Scientific Co. Ltd., Wonju, Republic of Korea) at 7000 rpm for 1 min, and then leached for 30 min. After the supernatant was filtered through filter paper, a test solution was prepared by adjusting it to slightly acidic (pH 5.5) using 5% sulfuric acid (H_2_SO_4_). After tilting the Conway dish slightly, 1 mL of 0.01 N H_2_SO_4_ was added to the inner shell, and 1 mL of the prepared test solution and saturated K_2_CO_3_, respectively, were added to the outer shell. The lid was closed, covered with airtight material, and fixed with a clip. The dish was then allowed to stand at 25 °C for 1 h. After completion of the reaction, 10 μL of Brunswik’s reagent was added to the H_2_SO_4_ solution in the inner shell and titrated with 0.01 N NaOH. TVB-N (mg/%) was calculated.

TVB-N (mg%) = 0.14 × ([*b* − *a*] × *f*)/*W* × 100 × 25 (3)

where *b* is the volume (mL) of 0.01 N NaOH consumed during titration in the blank test, *a* is the volume (mL) of 0.01 N NaOH consumed during titration of the test solution, *f* is the factor of 0.01 N NaOH, and *W* is the amount of sample taken (g).

#### 2.4.3. Total Viable Count (TVC)

TVC was measured using the dry film method [27]. Ten grams of mackerel were collected, put in a stomacher bag, mixed with sterile peptone saline buffer (Difco, Sparks, MD, USA), and homogenized for 1 min using a stomacher (BagMixer^®^ 400 CC, Interscience, Saint-Nom-la-Bretèche, France). After appropriately diluting, 1 mL of each dilution solution was inoculated on three sheets of dry film medium, and the number of red colonies was measured after 48 h 35 °C in an incubator. TVC was calculated.

(4) TVC(log⁡CFU/g)=dilution factor×number of red colonies

### 2.5. Statistical Analysis

A two-way analysis of variance (ANOVA) was used to examine differences in the effects of independent variables (ammonia gas concentration and temperature) on a dependent variable (indicator color). A three-way ANOVA was used for the effects of independent variables (storage time and temperature before use and binary value for presence/absence of light) on a dependent variable (indicator color). Tukey’s multiple comparison test was performed where significant differences were obtained by one-way ANOVA. All statistical analyses were conducted using IBM SPSS Statistics Version 20 (SPSS, Inc., Chicago, IL, USA).

## 3. Results and Discussion

### 3.1. Optimization of PCL/RCA NF Electrospinning Conditions

NF has an optimal microstructure when electrospun under steady-state conditions [28,29]. That is, there should be no beads or droplets in the structure of the NF. The steady state is determined by the physical and chemical properties of the solvent and solute (dielectric constant of the solvent, surface tension, viscosity, density of the solution) and the electrospinning process conditions (applied voltage) [29,30]. In this study, the FA/AA ratio and the contents of PCL and RCA were varied to establish a steady state. Electrospinning was performed for 30 min. As the electrospinning time increases, the thickness increases [31]. The magnitude of the color change depends on the concentration of anthocyanin and is independent of the thickness of the nanofiber. This is because in opaque materials such as nanofibers, the visible part is the surface layer, but the inside is hidden. Electrospinning times shorter than 30 min made it difficult to produce indicators because the nanofibers were too thin. 

#### 3.1.1. FA/AA Mixing Ratio

Compared to AA alone, when AA and FA are mixed, electrospinning can be performed in a steady state [32]. This is because FA, a polar solvent, can enhance electrical conductivity compared to AA.

**Figure 2:**
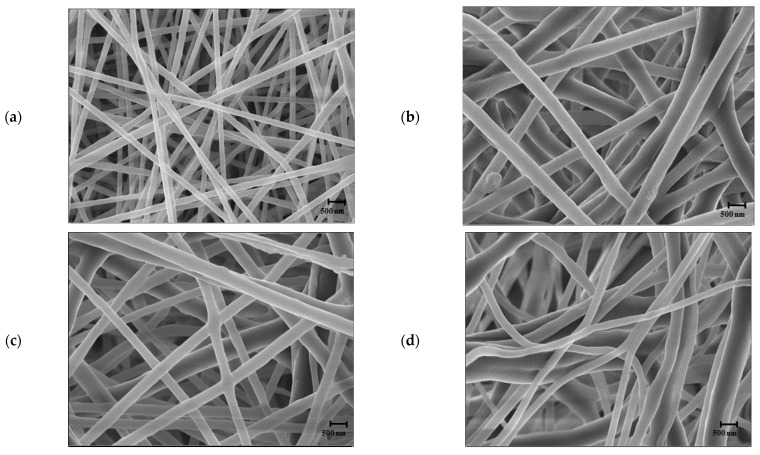
SEM micrographs of PCL/RCA (5:1) fibers electrospun at different bi-solvent ratios of FA/AA (*w*/*w*): (**a**) 7:3, (**b**) 5:5, (**c**) 3:7, and (**d**) 1:9 (PCL/bi-solvent: 15:85). The scale bars represent 500 nm.

The SEM micrographs of the electrospun NF by FA/AA solvent ratio are shown in Figure 2.

At the ratio of 7:3, the fine and straight fibers showed a dense and orderly arrayed microstructure, but as the content of FA decreased, the structure changed to a disorderly array of thick and bent fibers. Thus, the higher the FA content, the more improved the microstructure of NF. However, when the FA/AA ratio was as high as 9:1, NF was not formed and sprayed (the result of NF could not be presented). The fiber diameter increased from 185.60 ± 37.65 nm at the FA/AA ratio of 7:3 to 374.02 ± 113.33 at 5:5 and 473.21 ± 250.55 nm at 1:9.

The higher the FA content, the thinner the fiber. There are two reasons for this. One reason is that FA has a larger dielectric constant (57.2 at 298 K) than AA (6.6 at 298 K) [28]. The smaller the dielectric constant, the less instability in the jet during the electrospinning process. This shortens the length of the jet and increases the diameter of the fiber [29]. The second reason is that FA has a higher electrical conductivity than AA. As the electrical conductivity decreases, the diameter of the fiber increases [28]. When the mixing ratio was 9:1, it seems NF was not formed because of the large polarity and large dielectric constant of FA. High polarity and dielectric constant affect the electric field, causing the rearrangement of solvent molecules and destabilizing electrospinning [22,28,32]. Therefore, as a solvent to be used in this experiment, FA/AA at a ratio of 7:3 was selected, which led to the thinnest average diameter of NF and did not show beads.

#### 3.1.2. PCL Content

**Figure 3:**
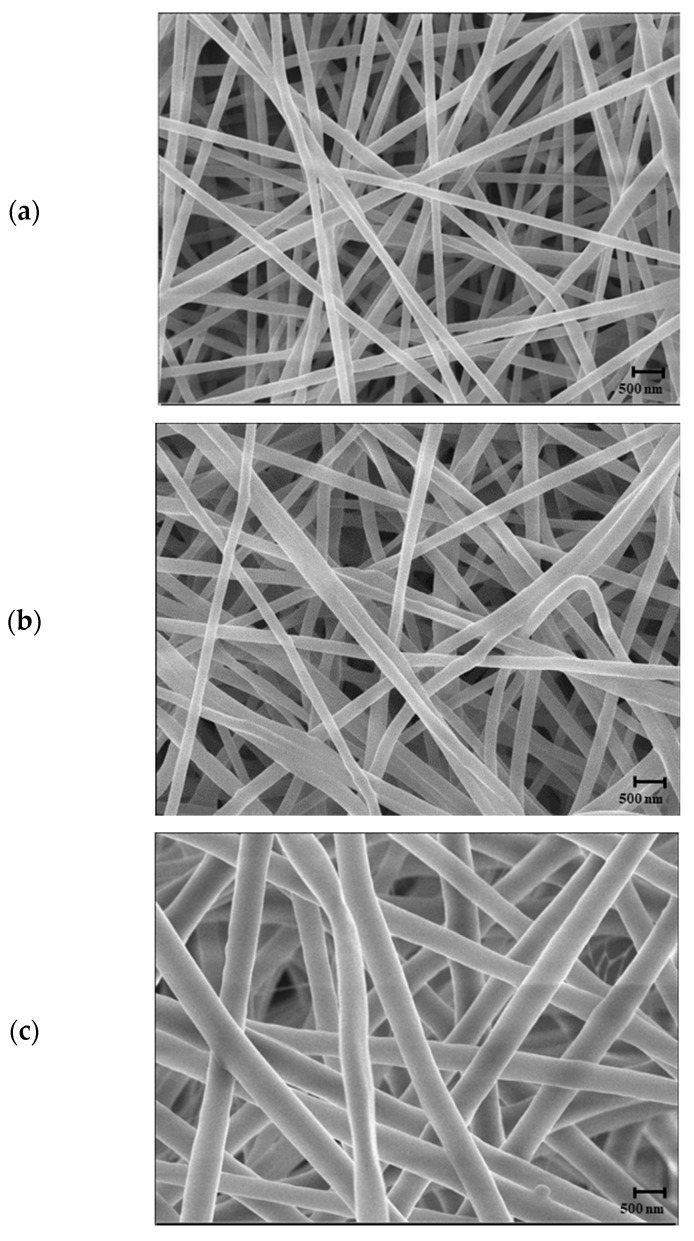
SEM micrographs of PCL/RCA (5:1) fibers electrospun at different concentrations of PCL (*w*/*w*): (**a**) 15%, (**b**) 17.5%, and (**c**) 20% (PCL/bi-solvent: 15:85, 17.5:82.5, and 20:80). The scale bars represent 500 nm.

The SEM micrographs of electrospun NF according to the content of PCL polymer are shown in Figure 3.

At the content of 15%, thin and straight fibers showed a dense and orderly microstructure, but as the content of PCL increased, thick and dense fibers formed an orderly arrangement structure. Thus, the microstructure of NF was improved as the content of PCL decreased. However, at the lowest examined PCL content of 12.5%, NF was not formed and sprayed. The fiber diameter electrospun from 15% PCL was 185.60 ± 37.65 nm, and NF with a diameter of 197.28 ± 100.27 and 496.30 ± 67.62 nm was electrospun from 17.5% and 20% PCL, respectively. The higher the PCL content, the shorter the dissolved state of the polymer is maintained during electrospinning, resulting in shorter stretches of the jet and faster solidification to produce thick NF [28], which are usually highly entangled [22]. However, a PCL content of 12.5% was too low and therefore electrosprayed, not electrospun. Therefore, the optimal polymer content was chosen as 15%, resulting in the thinnest average diameter of NF and no beads.

#### 3.1.3. RCA Content

**Figure 4:**
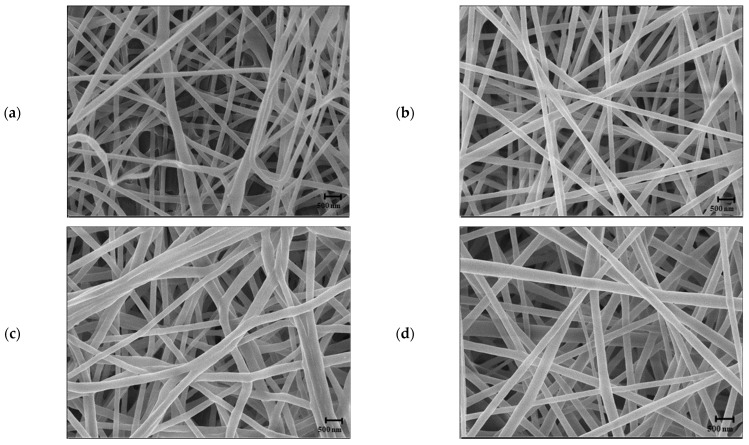
SEM micrographs of PCL/RCA fibers electrospun at different ratios of PCL/RCA (*w*/*w*): (**a**) 10:1, (**b**) 10:2, (**c**) 10:3, and (**d**) 10:4 (PCL/bi-solvent: 15:85). The scale bars represent 500 nm.

The SEM micrographs of NF according to the RCA content used as a color-changing substance are shown in Figure 4.

The microstructure changed from thin, straight fibers that were relatively randomly arranged for the 10% RCA NF to a structure in which thick, straight fibers were arranged in an orderly manner as the RCA content increased. It showed that the higher the RCA content, the larger the NF diameter. The fiber diameter measurements were 175.23 ± 27.60, 185.60 ± 37.66, 235.34 ± 60.37, and 305.92 ± 75.41 nm at 10, 20, 30, and 40% RCA, respectively. It also produced(showed) the same trend as Maftoonazad and Ramaswamy [33], that thicker fibers could be produced by the incorporation of red cabbage extract to poly(vinyl alcohol) NF. Similar to the reason for the increase in diameter with increasing PCL content, with the increase in solute content, the jet appears to have solidified quickly during electrospinning, shortening the stretching and eventually increasing the diameter. When NF is applied to a sensor, the smaller the diameter, the larger the surface area. Therefore, the lower the RCA content, the better [34]. For this reason, the 10 and 20% RCA NF with relatively small diameters were explored for their color properties.

As the RCA content increased, a clearer pink color was shown; the L*-value (lightness) and the b*-value (yellowness) decreased; and the a*-value (redness) increased (Table 1). Although 10% RCA NF showed the thinnest diameter, 20% RCA NF was selected as optimal because 20%, which did not have a large difference in diameter, showed a more vivid color.

### 3.2. Characteristics of PCL/RCA/NF Indicator

#### 3.2.1. Thickness and Porosity

Thinner and more porous indicators are more desirable for responsiveness because of their rapid reaction with the reactants [34]. The thickness was measured as 0.035 ± 0.002 mm, which was thinner than that of the PCL/anthocyanin indicator reported by Liu et al. [2] of 0.056 ± 0.020 mm. The porosity was calculated to be 85 ± 0.2%, which was higher than that of the PCL electrospun NF using only AA as a solvent reported by Ferreira et al. [23] as 82 ± 1%.

#### 3.2.2. FTIR

**Figure 5:**
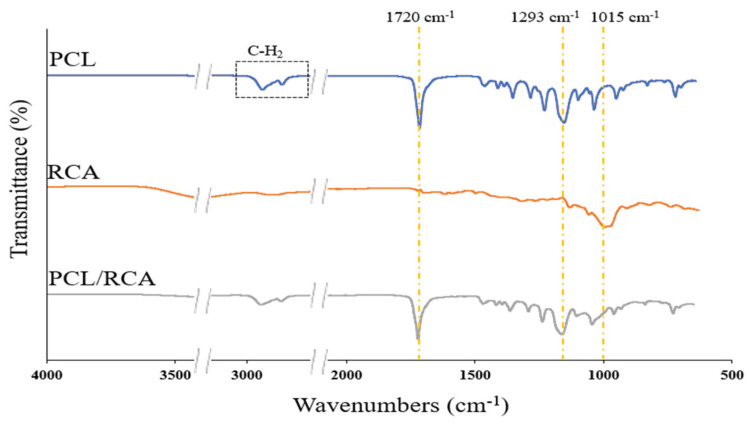
FT-IR spectra in expanded form in the ranges of 3300 to 2500 cm^−1^ and 2000 to 500 cm^−1^ for PCL, RCA, and PCL/RCA indicators.

FA/AA hydrolyzes the ester bond in PCL, and RCA has a relatively stable structure in acid, but it is known that denaturation may occur [10,35,36]. Whether FA/AA denatured PCL and RCA during electrospinning was analyzed by ATR FTIR spectroscopy. In the spectrum of PCL/RCA NF, distinct peaks appeared at 2936–2862, 1720, 1293, and 1015 cm^−1^, and several groups of peaks appeared in the range of 1461–959 cm^−1^ (Figure 5). In the typical spectrum of PCL, the C-H_2_ bond appears at 2930–2631 cm^−1^, the C=O bond at 1720 cm^−1^, C-C at 1469 cm^−1^, the C-O-C bond at 1294 cm^−1^, and C-O at 1293 cm^−1^ [37]. In the typical spectrum of RCA, the C=C bonds of the aromatic structure appear at 1650 and 1455 cm^−1^, the aromatic ring structure of C-H at 1015 cm^−1^, and the typical pyran ring of flavonoid compounds exhibits a peak at 1233 cm^−1^ [33]. The characteristic peaks at 2936–2862, 1720, and 1293 cm^−1^ of the PCL/RCA NF were consistent with the characteristic peaks of PCL, and the peaks at 1455 and 1015 cm^−1^ were consistent with the characteristic peaks of RCA. Therefore, it is judged that FA/AA did not affect the structure of PCL and RCA because the characteristic peaks in the spectrum of each sample were the same during FTIR analysis.

#### 3.2.3. Color Response to Ammonia Solution Vapor

**Figure 6:**
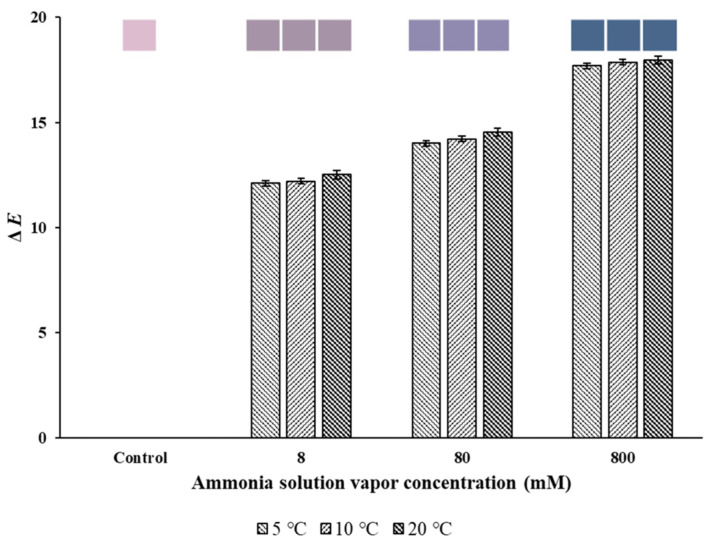
Total color value (Δ*E*) and images of the PCL/RCA/NF indicator after 30 min exposure to ammonia vapor at different temperatures. The error bars represent standard deviation (*n* = 3).

Colorimetric indicators for the detection of volatile amine from chicken breast and shrimp were exposed to 800 mM ammonia solution to evaluate their color changes [25]. Therefore, to evaluate the performance of the electrospun indicator in the current study, the indicator was exposed to the saturated gas of ammonia solutions of 8, 80, and 800 mM. In addition, experiments were conducted at 5, 10, and 20 °C to confirm the stability of color change with respect to temperature, which is an environmental variable that could affect the indicator color response. When exposed to the saturated gas of ammonia solution of 8 mM, the indicator was purple, but as the concentration of the ammonia solution increased, the color changed from purple to blue. Although the Δ*E* increased significantly (*p* < 0.05) with the increase in NH_3_ concentration (Figure 6), there was no significant difference (*p* > 0.05) according to temperature (Table 2). Furthermore, because of the lack of a significant interaction (*p* > 0.05) between the ammonia solution concentration and temperature, it was found that the color change in the indicator to the difference in NH_3_ gas concentration was not affected by temperature.

The color change in the indicator from pink to purple after exposure to ammonia is due to the pH change due to the inflow of ammonia gas. As the pH increases with exposure to increasing concentrations of ammonia gas, the color of RCA changes. In addition, the endpoint is dependent on the ammonia concentration because of the pH dependence of NH_3_ concentration [1,7].

Liu et al. [2] developed a PCL/anthocyanin electrospun indicator that changes color from purple to blue, while the indicator developed in this study changes from pink to blue. Therefore, the visibility of the color change was further enhanced after exposure to ammonia. FA has a lower p*K_a_* value (3.75) than AA (4.76) [38]. Consequently, it seems that the initial color of RCA changed from blue to pink as the p*K_a_* value of the solvent became lower and more acidic when FA was added.

#### 3.2.4. Stability during Storage

**Figure 7:**
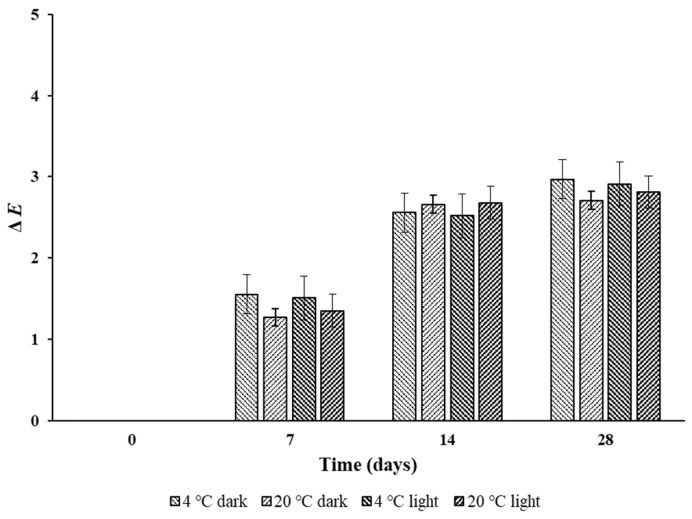
Total color change value (Δ*E*) during storage at 4 and 20 °C, 50% relative humidity, and with and without light. The error bars represent standard deviation (*n* = 3).

For RCA-based indicators, it is important to reduce color loss during storage as much as possible [39]. Therefore, to confirm whether the indicator changes color under the influence of temperature and light during storage before use, it was stored for 28 days at 20 and 4 °C under light and dark conditions. The Δ*E* value of the indicator according to storage days is shown in Figure 7. The maximum Δ*E* value was 3.7. It should be noted that a Δ*E* < 5 cannot be easily observed by the naked eye [40]. Bao et al. [41] reported that the Δ*E* value increased to 4.3 when an anthocyanin indicator prepared by the sol-gel method was stored for 14 days in the same environment condition. This was a greater change during a shorter period than when stored before use for 28 days in the current study. Therefore, within 28 days of storage, the color change in the indicator was not visible to the naked eye. Table 3 shows the results of analyzing the difference in color change according to time, temperature, and presence of light by three-way ANOVA. Only the color difference according to time was significant (*p* < 0.05), not the color difference according to temperature and light (*p* > 0.05). Within this, it was found that the color change in the indicator was stable as it was not affected by temperature and the presence or absence of light.

### 3.3. Application of Indicator

**Figure 8:**
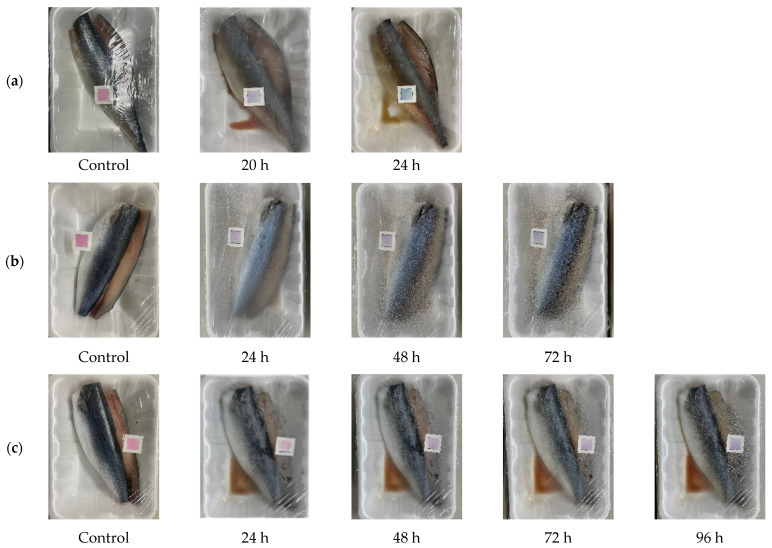
Color change in the indicators in mackerel packaging over time: (**a**) 20 °C, (**b**) 15 °C, and (**c**) 10 °C. Cloudy areas on the packaging wrap indicate water vapor condensed inside.

Distribution/storage temperatures for mackerel packaging may apply up to the freezing point [42]. However, in this study, higher temperatures (10, 15, and 20 ℃) were applied, which may correspond to temperature abuse that may occur during distribution/storage. The major role of the indicator is to monitor spoilage that may occur in these undesirable situations. The developed PCL/RCA indicator was attached to the packaging of mackerel to monitor the color change in the indicator (Figure 8). Changes in mackerel quality were confirmed via TVB-N and TVC as representative variables. These changes and the indicator chromaticity values are shown in Table 4. Lee et al. [43] reported mackerel was considered spoiled if the TVB-N content was 25 mg% or more. For TVC, 10^5^ or less is considered fresh fish meat, 10^5^ to 10^6^ is considered early spoilage, and 1.5 × 10^6^ CFU/g or more is considered spoiled [44].

At 20 °C, the content of TVB-N at 20 h was 27.07 ± 0.66 mg%, the TVC was 6.10 ± 0.02 log CFU/g, showing that spoilage had started, and the color of the indicator changed rapidly from pink to purple, resulting in a Δ*E* value of 7.27 ± 0.26. After storage for 24 h, the Δ*E* value was 14.01 ± 0.12, the indicator showed a blue color, and the TVB-N and TVC values were 30.07 ± 0.66 mg% and 6.80 ± 0.08, respectively, indicating that the fish was already spoiled. After storage at 15 °C for 24 h, the TVB-N value was 25.67 ± 0.66%, and the TVC value was 4.61 ± 0.02. Although TVC had not yet reached the limit of acceptability, the recommended TVB-N standard value for fresh fish had been exceeded, confirming that the fish had spoiled. At this time, the Δ*E* value was 8.86 ± 0.21. At 48 h, TVB-N and TVC met the spoilage criteria, and Δ*E* was 10.84 ± 0.38. At 10 ℃, the TVB-N content at 48 h was 25.67 ± 0.66 mg%, and the TVC was 5.54 ± 0.05. The mackerel was spoiled based on the TVB-N standard, and the Δ*E* value was 7.59 ± 0.14. At 72 h, both TVB-N and TVC had exceeded their acceptability limits, and Δ*E* was 8.29 ± 0.58.

From these results, the color change at each temperature was identifiable by the naked eye because the Δ*E* value was 5 or more and was highly related to the mackerel spoilage. The color changed from pink to purple, and as spoilage progressed, the indicator turned to the final blue. As discussed above, the color of RCA is dependent on the ammonia concentration. In the case of mackerel, the purple color of the indicator, just before the final blue, was set as the endpoint of fish freshness. As mackerel deteriorates during storage, its pH increases, which reflects the production of ammonia [42]. In the same manner, the pH of the indicator in the mackerel packaging also increases and changes color.

## 4. Conclusions

The NF microstructure and color change visibility of the conventional electrospun PCL/anthocyanin indicator using a single solvent (AA) could be improved by applying a bi-solvent (AA/FA). When electrospinning uses only AA, beads are formed on NF, and reproducibility is insufficient. To solve this problem, the performance of the indicator was optimized in terms of microstructure and color visibility using AA mixed with FA. An indicator with a smaller NF diameter and an initial pink color was produced compared to conventional indicators. When the indicator was exposed to various concentrations of amine gas and various temperatures, it showed different endpoints (purple and blue) depending on the concentration of amine gas regardless of the temperature. In addition, when applied to mackerel, one of the representative marine products, it was proved that freshness can be judged with the naked eye by showing a color change in the indicator from pink to purple. This indicator is expected to be used in other foods where volatile amines are a quality factor.

## Figures and Tables

**Table 1:** Mean fiber diameter, images, and color parameters of PCL/RCA fibers electrospun at different ratios of PCL/RCA.

Parameter	PCL/RCA (*w*/*w*)			
	10:01	10:02	10:03	10:04
Mean fiber diameter (nm)	175.2 ± 27.6 ^a^	185.6 ± 37.7 ^ab^	235.3 ± 60.4 ^b^	305.9 ± 75.4 ^c^
Indicator color	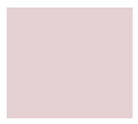	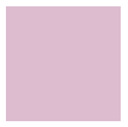	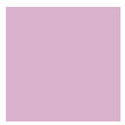	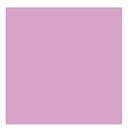
*L*	91.6 ± 0.2 ^a^	89.7 ± 0.4 ^b^	87.2 ± 0.1 ^c^	84.2 ± 0.2 ^d^
*a*	6.3 ± 0.1 ^a^	7.5 ± 0.1 ^b^	9.8 ± 0.1 ^c^	11.3 ± 0.1 ^d^
*b*	1.3 ± 0.01 ^a^	0.9 ± 0.1 ^b^	0.4 ± 0.2 ^c^	−0.1 ± 0.1 ^d^

^a–d^ Mean ± standard deviation (*n* = 3). Means with the same uppercase letter on the same row do not differ significantly (*p* > 0.05).

**Table 2:** Two-way ANOVA results of total color change (Δ*E*) of indicator for ammonia solution concentration and temperature.

Source	Degree of Freedom	Sum of Squares	Mean Squares	F-Value	*p*-Value ^a^
Ammonia solution concentration (A)	2	180.354	90.177	1120.777	0
Temperature (B)	2	0.43	0.215	2.674	0.096
Interaction (A × B)	4	0.614	0.153	1.908	0.153
Error	18	1.448	0.08		

^a^ Error probability (*n* = 3).

**Table 3:** Three-way ANOVA results of total color change (Δ*E*) of indicator for temperature, with and without lightness, and time.

Source	Degree of Freedom	Sum of Squares	Mean Squares	F-Value	*p*-Value ^a^
Temperature (A)	1	0.01	0.01	0.681	0.415
Lightness (B)	1	0.084	0.084	5.781	0.022
Time (C)	3	61.876	20.625	1416.538	0
Interactions:					
A × B	1	0	0	0.012	0.915
A × C	3	0.005	0.002	0.118	0.949
B × C	3	0.224	0.075	5.123	0.005
Total interaction	3	0.012	0.004	0.26	0.85
Error	32	0.466	0.015		

^a^ Error probability (*n* = 3).

**Table 4:** Total color change (Δ*E*) and quality factors of mackerel over time.

Temperature (°C)	Time (h)	TVB-N ^b^ (mg%)	TVC ^c^ (log CFU/g)	Δ*E*
20	0	13.07 ± 0.66 ^a^	3.59 ± 0.56	-
	20	27.07 ± 0.66	6.10 ± 0.02	7.27 ± 0.26
	24	30.07 ± 0.66	6.80 ± 0.08	14.01 ± 0.12
15	0	13.07 ± 0.66	3.59 ± 0.06	-
	24	25.67 ± 0.66	4.61 ± 0.02	8.86 ± 0.21
	48	30.8 ± 1.14	6.45 ± 0.01	10.84 ± 0.38
	72	41.07 ± 1.31	7.43 ± 0.03	11.16 ± 0.08
10	0	13.07 ± 0.66	3.59 ± 0.06	-
	24	21.93 ± 0.66	4.54 ± 0.05	4.89 ± 0.31
	48	25.67 ± 0.66	5.54 ± 0.05	7.59 ± 0.14
	72	29.87 ± 0.66	6.12 ± 0.03	8.29 ± 0.58
	96	39.67 ± 0.66	7.16 ± 0.01	10.41 ± 0.21

^a^ Mean ± standard deviation (*n* = 3). ^b^ Total volatile basic amines. ^c^ Total viable cells.

## Data Availability

The data presented in this study are available upon request from the corresponding author.
